# Etymology of Cholera

**DOI:** 10.3201/eid1803.111636

**Published:** 2012-03

**Authors:** Antonis A. Kousoulis

**Affiliations:** University of Athens, Athens, Greece; and Society of Junior Doctors, Athens

**Keywords:** cholera, etymology, Hippocrates, cholē, cholēdra, cholās

**To the Editor:** I read with great interest the article by Männikkö (*1*) on the etymology of cholera. However, discovering the origin of the word with certainty is an intricate matter. The word cholera is undoubtedly Greek because Hippocrates was the first to mention it in his writings, although the exact disease he was referring to is unknown (*2**,**3*).

Apart from the rather probable derivation from *cholē* (the word for bile and a dominant term in the humoral theory, which is of Hippocratic and not Galenic [*1*] provenance), one more hypothesis has been suggested. The word *cholera*, sometimes *cholēdra*, originally meant a gutter (*4*). Following this connection, cholera came to mean a pestiferous disease during which fluids are forcefully expelled from the body, resembling a gutter (*4*). This etymology-derived definition could suggest that Hippocrates and Galen, the prolific medical writers of antiquity who each in his time referred to cholera, may have witnessed cases of this infectious disease, albeit not in the epidemic form it took in ancient India (*5*).

In addition, a missing clue on this issue is that cholera might derive from *cholās*, an Attic word meaning intestine, which has not survived in modern Greek (*4*). This new connection with the gastrointestinal tract further suggests possible knowledge of cholera in its present form, mainly diarrhea and vomiting. Hippocrates made such a reference, although loosely (*2*). Reaching a conclusion on the etymology of cholera remains intriguing.
